# Acute Cardiovascular Events after Herpes Zoster: A Self-Controlled Case Series Analysis in Vaccinated and Unvaccinated Older Residents of the United States

**DOI:** 10.1371/journal.pmed.1001919

**Published:** 2015-12-15

**Authors:** Caroline Minassian, Sara L. Thomas, Liam Smeeth, Ian Douglas, Ruth Brauer, Sinéad M. Langan

**Affiliations:** 1 Faculty of Epidemiology and Population Health, London School of Hygiene & Tropical Medicine, London, United Kingdom; 2 Department of Health Services and Population Research, Kings College London, London, United Kingdom; The George Institute for Global Health, AUSTRALIA

## Abstract

**Background:**

Herpes zoster is common and can have serious consequences. Additionally, emerging data suggest an increased risk of acute cardiovascular events following herpes zoster. However, to our knowledge, existing association studies compare outcomes between individuals and are therefore vulnerable to between-person confounding. In this study, we used a within-person study design to quantify any short-term increased risk of acute cardiovascular events (stroke and myocardial infarction [MI]) after zoster and to assess whether zoster vaccination modifies this association.

**Methods and Findings:**

The self-controlled case series method was used to estimate rates of stroke and acute MI in defined periods after herpes zoster compared to other time periods, within individuals. Participants were fully eligible Medicare beneficiaries aged ≥65 y with a herpes zoster diagnosis and either an ischemic stroke (*n* = 42,954) or MI (*n* = 24,237) between 1 January 2006 and 31 December 2011. Age-adjusted incidence ratios (IRs) for stroke and MI during predefined periods up to 12 mo after zoster relative to unexposed time periods were calculated using conditional Poisson regression. We observed a marked increase in the rate of acute cardiovascular events in the first week after zoster diagnosis: a 2.4-fold increased ischemic stroke rate (IR 2.37, 95% CI 2.17–2.59) and a 1.7-fold increased MI rate (IR 1.68, 95% CI 1.47–1.92), followed by a gradual resolution over 6 mo. Zoster vaccination did not appear to modify the association with MI (interaction *p*-value = 0.44). We also found no evidence for a difference in the IR for ischemic stroke between vaccinated (IR 1.14, 95% CI 0.75–1.74) and unvaccinated (IR 1.78, 95% CI 1.68–1.88) individuals during the first 4 wk after zoster diagnosis (interaction *p*-value = 0.28). The relatively few vaccinated individuals limited the study’s power to assess the role of vaccination.

**Conclusions:**

Stroke and MI rates are transiently increased after exposure to herpes zoster. We found no evidence for a role of zoster vaccination in these associations. These findings enhance our understanding of the temporality and magnitude of the association between zoster and acute cardiovascular events.

## Abstract 2

Using U.S. Medicare data, Caroline Minassian and colleagues test for associations between shingles and subsequent myocardial infarction or stroke.

## Editors' Summary

**Background:**

Chickenpox, which is caused by the varicella zoster virus, is a common childhood infectious disease. Although recovery from chickenpox is usually quick, the varicella zoster virus can persist throughout life inside the nervous system. The dormant virus causes no symptoms, but if it becomes activated, it causes herpes zoster (also known as shingles or zoster), a painful skin rash. Anyone who has had chickenpox can develop zoster, but the condition is most common and most severe in elderly people—half of zoster episodes occur in people aged more than 60 years. Early signs of zoster include burning or shooting pain, tingling, and itching. Blister-like sores, which last 1–14 days, then develop on one side of the face or on one side of the body in a horizontal band. The pain of zoster, which can be debilitating, can continue for years after the rash disappears—a condition called post-herpetic neuralgia, which greatly reduces the quality of life. There is no cure for zoster, but vaccination can prevent the condition or lessen its effects, and early treatment with antivirals may prevent lingering pain by inhibiting viral replication.

**Why Was This Study Done?:**

Association studies suggest that people have an increased risk of an acute cardiovascular event—a stroke or a myocardial infarction (MI; heart attack)—in the weeks and months following a zoster episode. However, these studies mainly compared the incidence of stroke and MI (the number of strokes and MIs in a given time period) among people who had had zoster with the incidence among people who had not had zoster and were therefore vulnerable to between-person confounding. That is, rather than zoster increasing a person’s risk of MI or stroke, people who had had zoster may have shared another unknown characteristic (confounder) that was responsible for their increased risk of MI and stroke. Here, the researchers investigate whether there is an increased risk of acute cardiovascular events after zoster using a self-controlled case series study design. Specifically, they compare the incidence of MI and ischemic stroke in a population of individuals during a period of time before and more than a year after the individuals had zoster with the incidence of MI and ischemic stroke in the same individuals during the weeks and months right after they were diagnosed with zoster. Because each case is his/her own control, between-person confounding is avoided.

**What Did the Researchers Do and Find?:**

The researchers identified 42,954 Medicare beneficiaries aged ≥65 years who had had a zoster diagnosis and an ischemic stroke and 24,237 beneficiaries who had had a zoster diagnosis and an MI during a five-year period (Medicare is a US government health insurance plan that mainly covers the healthcare costs of elderly individuals; an ischemic stroke occurs when a blood clot blocks the brain’s blood supply). They then calculated age-adjusted incidence ratios for stroke and MI during predefined periods up to 12 months after a diagnosis of zoster relative to the time period before and more than a year after a diagnosis of zoster (the “baseline” period). Compared to the baseline period, there was a 2.4-fold increased rate of ischemic stroke and a 1.7-fold increased rate of MI in the first week after zoster. The increased rate of acute cardiovascular events reduced gradually over the six months following zoster. There was no evidence that MI or ischemic stroke incidence ratios differed between individuals who had been vaccinated against zoster (but still contracted the disease) and those who had not been vaccinated.

**What Do These Findings Mean?:**

These findings suggest that zoster is associated with transiently increased rates of ischemic stroke and MI. Zoster vaccination—which reduces zoster incidence by half and can reduce zoster symptoms—was not found to modify the associations between zoster and ischemic stroke or MI. However, this second finding needs confirmation: the study had limited power to detect an effect of vaccination on ischemic stroke or MI following zoster, given the small number of participants who were vaccinated and then developed zoster despite being vaccinated. Another potential study limitation is residual confounding. Although the self-controlled case series design controls for time-fixed confounders, time-varying factors such as major life events or stress may still play a role in the association. Thus, an episode of zoster may cause stress, which could then lead to an increased risk of MI or stroke. Importantly, however, these findings enhance our understanding of the magnitude and timing of the association between zoster and acute cardiovascular events and provide information that might help to prevent such events in older individuals.

**Additional Information:**

This list of resources contains links that can be accessed when viewing the PDF on a device or via the online version of the article at http://dx.doi.org/10.1371/journal.pmed.1001919.

- The US Centers for Disease Control and Prevention has detailed information about all aspects of shingles (zoster), including information on vaccination, and information on stroke and myocardial infarction
- The National Institutes of Health Senior Health website includes information on zoster, a video describing a personal experience of zoster, and information on stroke and heart attack
- The UK National Health Service Choices website provides information about all aspects of zoster and about cardiovascular disease
- MedlinePlus provides links to other resources about zoster, stroke, and heart attack (in English and Spanish)

## Introduction

Strong and increasing evidence supports a period of increased cardiovascular risk shortly after exposure to specific infections, with rates of myocardial infarction (MI) and stroke increased 5- and 3-fold, respectively, following acute respiratory infection [1]. The basis for increased cardiovascular events following acute infection is hypothesized to be endothelial dysfunction, characterized by atheromatous plaque rupture and the development of a prothrombotic environment [2]. As acute cardiovascular diseases (CVDs), specifically ischemic stroke and MI, are major causes of morbidity and mortality in the US and worldwide, understanding the basis for acute cardiovascular events and any potential for prevention becomes increasingly important [3].

Herpes zoster results from reactivation of dormant varicella zoster virus (VZV). Herpes zoster is an important disease as it affects 1 million Americans per year and is frequently complicated by prolonged, severe, disabling pain, a condition called post-herpetic neuralgia (PHN) [4,5]. Zoster-associated morbidity led to the introduction of a targeted vaccination program for individuals aged 60 y or greater in the US in 2006. The zoster vaccine has been shown to be effective in routine practice against incident zoster and PHN. Despite this, uptake of this vaccine has been disappointing (3.9%) following its introduction in the US, with important discrepancies in vaccine uptake by race and by income status [6].

Recent studies have proposed that the risk of stroke may be increased in the year following an acute episode of herpes zoster, possibly also mediated by VZV replication in arterial walls, resulting in cerebral vasculopathy [7]. Most studies assessing the association between herpes zoster and stroke have been limited by residual confounding because comparisons were made between individuals who developed herpes zoster and those who did not, and these populations have important differences in underlying vascular risk that are difficult to measure and account for. In a previous study, our research group used the self-controlled case series (SCCS) method, which eliminates between-person confounding, to demonstrate that there is an increased risk of stroke in the first 6 mo following herpes zoster in the UK population and that antiviral therapy might lessen this association [8]. One UK cohort study, which may be limited by residual confounding, also suggested a longer-term increased risk of stroke and MI up to 24 y following acute herpes zoster [9]. To our knowledge, no previous study has determined the risk of MI in the period immediately following herpes zoster or assessed the role of zoster vaccination in the association between zoster and acute cardiovascular events.

We used administrative claims data from older US Medicare beneficiaries to test the hypothesis of an increased risk of stroke and MI in periods shortly following episodes of herpes zoster and to assess whether zoster vaccination might modify this association.

## Methods

### Ethics

Ethics approval was obtained from the Centers for Medicare & Medicaid Services (CMS) (data use agreement 21520) and from the London School of Hygiene & Tropical Medicine ethics committee.

### Data Source

Medicare is a US government health insurance plan that mainly covers healthcare costs for individuals aged ≥65 y, serving 15% of the US population. Coverage includes inpatient and outpatient care, physician/preventive services, facility charges, and prescription drug benefits. This study used Medicare administrative claims data (research identifiable files including Medicare Provider Analysis and Review [inpatient hospital and skilled nursing facilities], outpatient, carrier [physician/supplier], and prescription drug claims) for the period 1 January 2006 to 31 December 2011, obtained from the CMS.

### Study Design

The SCCS method was used to estimate the rate of acute cardiovascular events (stroke and MI) in defined periods after herpes zoster diagnosis compared to other time periods, within individuals [10]. The SCCS approach overcomes the problem of between-person confounding inherent in other observational study designs by making within-person comparisons in individuals with zoster, whereby each case is his/her own control. Our null hypothesis was that vascular event rates remain constant from day to day and are not affected by exposure to zoster.

### Participants

The source population included all Medicare beneficiaries aged 65 y or older enrolled in Medicare Parts A (hospital insurance), B (supplemental medical insurance), and D (prescription drug coverage) who had both a zoster diagnosis and a CMS Chronic Conditions Data Warehouse flag for acute MI or stroke/transient ischemic attack (derived from the CMS’s own algorithms) during the study period.

Participants were observed from the date they fulfilled the following criteria: (i) 12 mo of continuous enrollment in Medicare Parts A and B, (ii) eligible for Medicare Part D, and (iii) not in a health maintenance organization (HMO) (claims for beneficiaries enrolled in HMOs are not processed by CMS; hence, their clinical data are incomplete). The criterion of 12-mo minimum enrollment in Medicare Parts A and B was applied to enable the study of incident rather than prevalent events. Observation was censored at the earliest of the following: (i) the participant died, (ii) the participant joined an HMO, (iii) the participant lost eligibility for Medicare Part A, B, or D, or (iv) the study period ended (31 December 2011).

We identified each participant’s earliest recorded zoster episode and vascular event during the observation period and excluded individuals with evidence of vascular events or herpes zoster prior to the observation period. Diagnoses of incident events were extracted from outpatient and carrier (healthcare provider) files and from the primary diagnostic field in inpatient files. Patients with only secondary inpatient diagnoses were excluded because of the uncertainty of the timing of zoster or cardiovascular events, since these events were not the primary reason for admission to hospital and hence may have occurred at any time during hospitalization. For the stroke analyses, we additionally excluded individuals with (i) subarachnoid hemorrhage or established risk factors for subarachnoid hemorrhage, including cerebral aneurysms in the circle of Willis or arteriovenous malformations, at any time during enrollment and (ii) encephalitis diagnoses recorded up to 12 mo after the stroke, which may represent encephalitis initially misclassified as stroke.

### Exposure

We defined incident herpes zoster as the presence of International Classification of Diseases, Ninth Revision, Clinical Modification (ICD-9-CM) diagnostic code 053*x* (where *x* indicates that the fourth/fifth digits can take any value, excluding PHN codes 05312 and 05313) with an antiviral prescription in the 7 d before or after diagnosis. The requirement for antiviral therapy has been shown to improve the positive predictive value of using zoster diagnosis codes to identify incident cases [11]. Herpes zoster ophthalmicus (HZO) was identified from ICD-9-CM code 0532*x* recorded up to 12 mo after the incident zoster code or, when zoster codes were nonspecific, from either (i) acute eye infections or associated treatments within 2 wk of the zoster diagnosis or (ii) specific non-acute eye conditions associated with zoster, e.g., conjunctival scarring or episcleritis, recorded for the first time up to 3 mo after zoster. Exposure to HZO was analyzed separately from exposure to herpes zoster as previous research has suggested a markedly increased risk of stroke in this group [12]. In keeping with the primary zoster definition, an accompanying antiviral claim was also required for HZO.

Herpes zoster vaccination status was ascertained from records of American Medical Association Current Procedural Terminology (CPT) code 90736 in carrier files or US Food and Drug Administration National Drug Codes for zoster vaccine purchase in participants’ Medicare Part D drug files. Additionally, vaccine administration records up to 7 d after purchase (CPT code 90471 or Healthcare Common Procedure Coding System code G0377) were identified. We estimated the date of zoster vaccination as the earlier of the date of CPT code 90736 or date of administration. In the absence of a corresponding administration date and when no CPT code 90736 was recorded, the vaccine purchase date was used as a proxy (procedure/administration dates were considered better estimates of vaccination date than purchase dates).

### Outcomes

We identified and classified acute cardiovascular events with specific ICD-9-CM codes for stroke (433*x*1 or 434*x*1 [ischemic], 431 or 4329 [hemorrhagic], or 436 [nonspecific]) and MI (410*x*, excluding 410*x*2 codes, which indicate a subsequent episode of care). As our primary interest was in acute thrombotic cardiovascular events (ischemic stroke and MI), we excluded hemorrhagic strokes from the primary analysis. To do this, we first designated stroke episodes such that successive stroke codes within 28 d of each other were assumed to correspond to the same episode. Then, for episodes containing stroke codes of different types, we used the following hierarchy for classification: hemorrhagic codes took precedence (based on the assumption that hemorrhagic strokes are coded more accurately than ischemic strokes), and ischemic codes trumped nonspecific codes. Because hemorrhagic strokes could potentially result from arterial rupture or aneurysm following VZV vasculopathy, or from a transient spike in blood pressure (e.g., in response to zoster-associated pain), we undertook (in response to peer review) a secondary analysis of hemorrhagic strokes. To maintain an important assumption of the SCCS method—that recurrent outcome events within individuals are independent—we restricted all analyses to participants’ first stroke or MI in the observation period.

### Comorbidities and Demographics

For descriptive purposes, preexisting CVD (MI, stroke, transient ischemic attack, ischemic heart disease, heart failure, or atrial fibrillation) and risk factors for CVD (hypertension, hyperlipidemia, diabetes, chronic kidney disease, or chronic obstructive pulmonary disease) were identified using CMS Chronic Conditions Data Warehouse flags indicating the earliest occurrence of each condition. The number of unique prescriptions individuals received in the 12 mo before their vascular event (as an indicator of overall poorer health) was determined by identifying all unique prescriptions and excluding duplicate records with the same product code or the same generic and brand names on the same date. Ethnicity was defined according to the Social Security Administration’s master beneficiary record (based on self-report) and categorized as white, black, Asian, Hispanic, and other/unknown. Participation in “state buy-in,” whereby the state pays Medicare premiums for low-income individuals, was used as a proxy for low income. Current age was derived from date of birth and was included in all analyses as a time-varying covariate.

### Analysis

**Fig 1:**
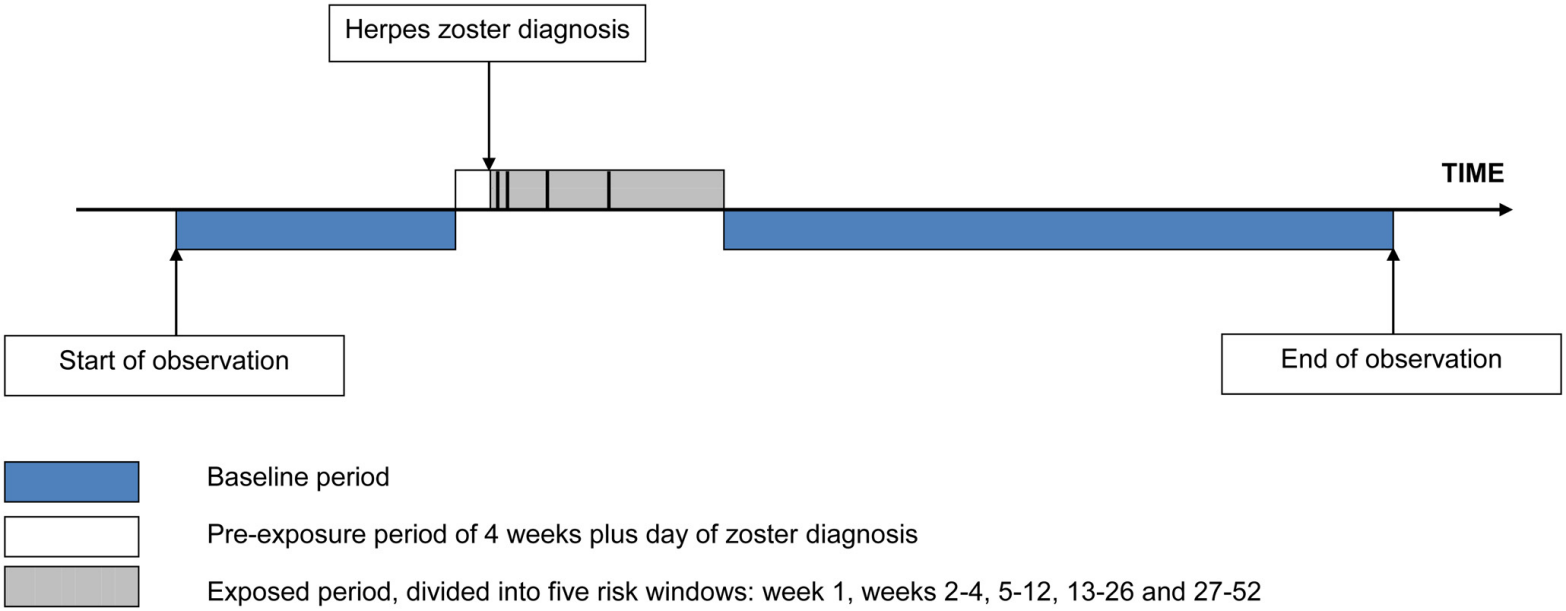
Pictorial representation of the self-controlled case series design, showing the observation period for a single individual.

Incidence ratios (IRs) for stroke and MI during predefined periods after exposure to zoster relative to unexposed time periods were calculated using conditional Poisson regression, adjusting for age in 2-y bands. We defined a 12-mo exposed period starting the day after zoster diagnosis, subdivided into five risk windows: week 1, weeks 2–4, 5–12, 13–26, and 27–52 after zoster diagnosis. When calculating the IR for any given risk window, the four remaining risk windows were still considered “exposed.” All other observation time made up the baseline (unexposed) period, with the exception of the day of and the 4 wk before zoster diagnosis. The day of zoster diagnosis was excluded because MI or stroke records on the same day as incident zoster may represent retrospective diagnoses. We excluded the 4 wk prior to zoster diagnosis because vascular risk during this pre-zoster period may be higher or lower than in other unexposed periods because the chances of developing and/or presenting with zoster shortly after a vascular event may differ from those of other time periods. Removing these periods from baseline was an attempt to avoid bias in the effect estimates that could have operated in either direction depending on the variation in vascular risk. Fig 1 illustrates the observation period for a single individual.

The primary analysis assessed the association between zoster (all cases, irrespective of site) and ischemic stroke and MI separately. Additionally, the association between HZO and each outcome was assessed. A secondary analysis of hemorrhagic strokes (excluded from the primary analysis) was conducted to examine the association between zoster and this stroke subtype. We undertook further analyses to explore whether vascular risk after zoster was different in those who received the zoster vaccine versus those who did not. We defined vaccinated individuals as those who received the zoster vaccine before they developed zoster. To allow for effective immunization, these individuals were classified as vaccinated from 30 d after receipt of the vaccine [13]. Hence, for these stratified analyses, the adjusted start date for vaccinated individuals was from the later of this date and their original observation start date. Unvaccinated individuals comprised those who did not receive the zoster vaccine and, additionally, those who were vaccinated after their incident zoster episode and whose observation was thus curtailed at vaccination. Because of the small numbers of individuals in the vaccinated strata, the first two post-zoster risk windows (week 1 and weeks 2–4) were combined.

The SCCS method requires that each individual’s observation period is independent of the event time [14]. For outcomes such as stroke or MI, which increase mortality in the short term, this assumption may be violated. To allow for nonrandom censoring of observation due to death as a result of a vascular event (which may lead to bias if uncorrected), an extension to the standard SCCS method was used [15]. The extension works by conditioning explicitly on the age at censoring in cases. Under this new model, the interpretation of the exposure effect is the same as for the standard model, but the age effect takes on a new interpretation, representing the combined effect of age-specific relative incidence and censoring. By introducing an additional term in the likelihood that depends on the censoring process, the independence assumption is removed. The extension has been shown to successfully correct for biased estimates in the presence of substantial post-event censoring due to mortality [16]. In addition, the primary analysis was repeated excluding individuals who died or whose observation ended within 90 d after their vascular event (possibly indicating death).

Standard SCCS analyses were undertaken using STATA, version 13 (StataCorp); modified analyses accounting for event-dependent censoring were performed in R, version 3.0.2.

## Results

**Fig 2:**
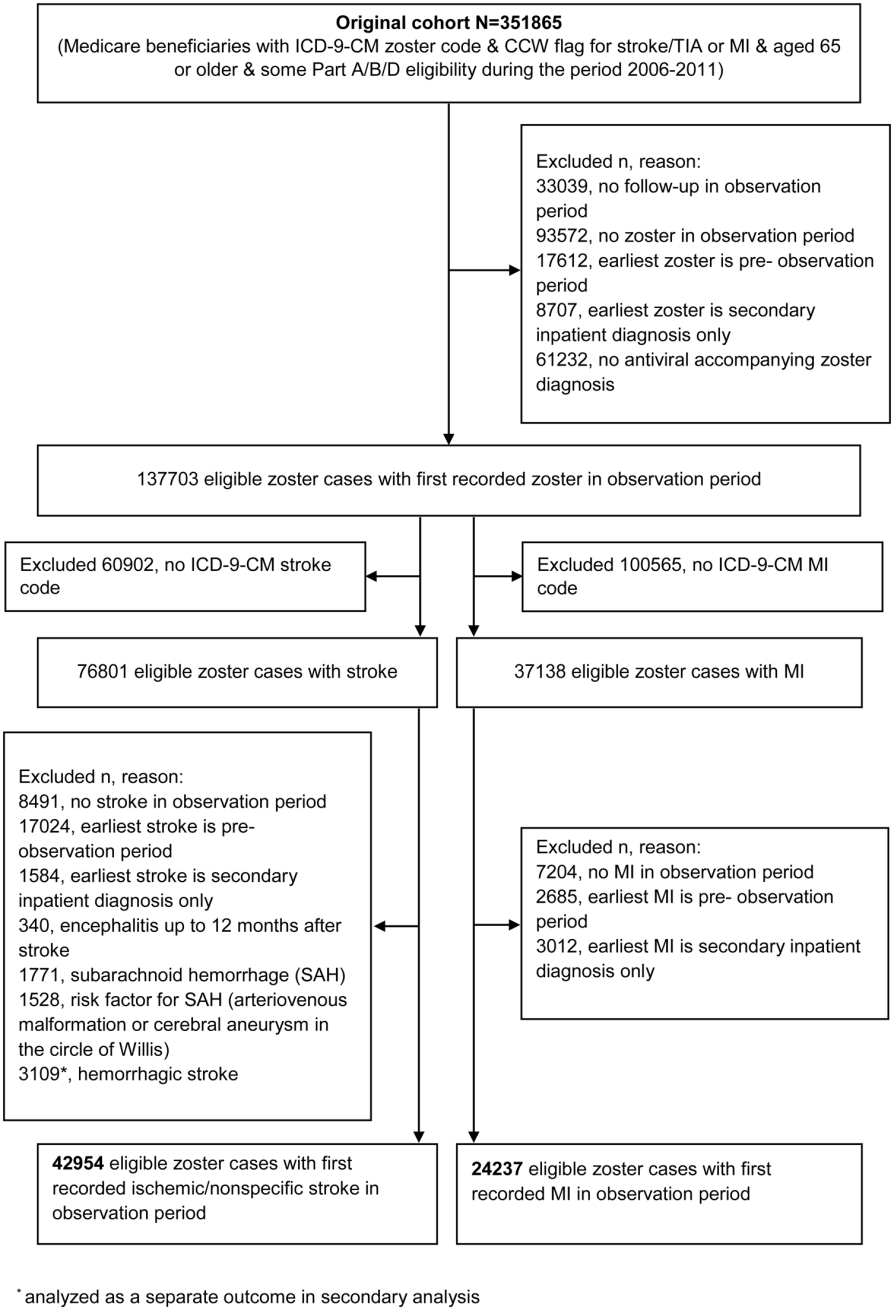
Identification of study participants. CCW, Chronic Conditions Data Warehouse; TIA, transient ischemic attack.

The initial study population comprised 351,865 individuals, of whom 42,954 zoster cases with incident ischemic stroke and 24,237 zoster cases with acute MI fulfilled the eligibility criteria and were included in the primary analysis (Fig 2). Characteristics of these individuals are presented in Table 1. The median age at zoster diagnosis was 80 y (interquartile range [IQR] 74–86 y), and the median observation period was 5 y (IQR 4–5 y). The majority of participants were female (71% of zoster cases with stroke, 64% of zoster cases with MI); 89% of participants were white (88% of strokes, 90% of MIs), 5% were black, and the remaining 6% were Asian (2%), Hispanic (2%), or of other/unknown ethnicity (2%). In all, 16% of zoster cases had HZO; the remaining 84% had zoster of an unspecified site. Also, 34% of cases were of low income, and 90% had evidence of preexisting CVD before zoster diagnosis.

**Table 1:** Participant characteristics.

Characteristic	Zoster Cases with Ischemic Stroke (*n* = 42,954)	Zoster Cases with MI (*n* = 24,237)
**HZO**	6,971 (16.2%)	3,946 (16.3%)
**Age at zoster diagnosis (years)**	80.4 (74.4–85.9)	79.7 (73.5–85.5)
65–69	4,330 (10.1%)	2,854 (11.8%)
70–79	16,354 (38.1%)	9,602 (39.6%)
80–89	17,568 (40.9%)	9,286 (38.3%)
≥90	4,702 (10.9%)	2,495 (10.3%)
**Age at HZO diagnosis (years)**	81.1 (75.0–86.4)	80.3 (74.2–86.2)
**Gender**		
Male	12,672 (29.5%)	8,640 (35.6%)
Female	30,282 (70.5%)	15,597 (64.4%)
**Ethnicity**		
White	37,943 (88.3%)	21,693 (89.5%)
Black	2,319 (5.4%)	1,151 (4.8%)
Asian	970 (2.3%)	461 (1.9%)
Hispanic	1,033 (2.4%)	555 (2.3%)
Other/unknown	689 (1.6%)	377 (1.5%)
**Low income** ^a^	14,742 (34.3%)	8,265 (34.1%)
**Number of prescriptions in 12 mo before vascular event**	48 (28–77)	51 (29–81)
**CVD before zoster** ^b^	38,496 (89.6%)	21,969 (90.6%)
**Risk factor for CVD before zoster** ^c^	42,216 (98.3%)	23,916 (98.7%)
**Total observation (years)**	5.0 (4.0–5.0)	5.0 (3.8–5.0)
**Zoster vaccination status**		
Vaccinated before zoster	1,213 (2.8%)	567 (2.3%)
Vaccinated after zoster^d^	2,759 (6.4%)	1,351 (5.6%)
Unvaccinated	38,982 (90.8%)	22,319 (92.1%)
**Died or follow-up ended ≤90 d after vascular event**	4,304 (10.0%)	3,986 (16.4%)

Data are given as *n* (percent) or median (IQR).

^a^State buy-in at any time during enrollment.

^b^MI, stroke, transient ischemic attack, ischemic heart disease, heart failure, or atrial fibrillation.

^c^Hypertension, hyperlipidemia, diabetes, chronic kidney disease, or chronic obstructive pulmonary disease.

^d^Includes 27 stroke cases and 19 MI cases who received vaccine on day of zoster diagnosis.

A small minority of cases received the zoster vaccine before developing zoster (3% of cases with stroke, 2% of cases with MI), 6% received the vaccine after zoster diagnosis, and 91% were unvaccinated throughout the observation period. Characteristics of individuals included in the analyses stratified by zoster vaccination status are given in S1 Table. Vaccinated and unvaccinated individuals had similar median age, gender, and preexisting CVD risk profiles, although unvaccinated individuals were more than twice as likely to be of low income and received more prescriptions in the year leading up to their vascular event compared to vaccinated individuals. Ethnicity data among vaccinated individuals could not be reported because some numbers were small enough to be restricted by the CMS small-sized-cell privacy policy.

The rate of ischemic stroke was significantly increased up to 3 mo after zoster diagnosis (any site) compared to the baseline rate: the most marked increase, 2.4-fold, was observed within the first week (IR 2.37, 95% CI 2.17–2.59), reducing to 1.6-fold in weeks 2–4 (IR 1.55, 95% CI 1.46–1.66), 1.2-fold in weeks 5–12 (IR 1.17, 95% CI 1.11–1.22), and resolving over the subsequent 3 mo (weeks 13–26: IR 1.03, 95% CI 0.99–1.07; weeks 27–52: IR 1.00, 95% CI 0.96–1.03). A similar though less marked association was observed for MI in the 3 mo after zoster diagnosis, with a 68% increased MI rate in the first week (IR 1.68, 95% CI 1.47–1.92) compared to baseline and a similar pattern of resolution as for stroke (weeks 2–4: IR 1.25, 95% CI 1.14–1.37; weeks 5–12: IR 1.07, 95% CI 1.00–1.14; weeks 13–26: IR 1.02, 95% CI 0.96–1.07; weeks 27–52: IR 1.02, 95% CI 0.98–1.07) (Table 2).

**Table 2:** Primary analysis: age-adjusted incidence ratios for ischemic stroke and myocardial infarction in risk periods after zoster diagnosis.

Risk Period	Number of Ischemic Stroke Cases (*n* = 42,954)	Ischemic Stroke IR^a^ (95% CI)	Number of MI Cases (*n* = 24,237)	MI IR^a^ (95% CI)
**Baseline**	32,179	1	18,071	1
**Risk period after zoster**				
1 wk	499	2.37 (2.17–2.59)^b^	213	1.68 (1.47–1.92)^b^
2–4 wk	967	1.55 (1.46–1.66)^b^	470	1.25 (1.14–1.37)^b^
5–12 wk	1,841	1.17 (1.11–1.22)^b^	1,019	1.07 (1.00–1.14)^c^
13–26 wk	2,588	1.03 (0.99–1.07)	1,537	1.02 (0.96–1.07)
27–52 wk	3,981	1.00 (0.96–1.03)	2,459	1.02 (0.98–1.07)

^a^IRs age-adjusted in 2-y bands.

^b^ *p <* 0.001.

^c^ *p <* 0.05.

Analyses restricted to cases with HZO (*n* = 6,971 with ischemic stroke, 3,946 with MI) yielded associations comparable to those of the primary analysis (week 1 after HZO diagnosis: stroke IR 2.73, 95% CI 2.22–3.35; MI IR 2.06, 95% CI 1.52–2.79) that resolved over the same time period (Table 3).

**Table 3:** Age-adjusted incidence ratios for ischemic stroke and myocardial infarction in risk periods after herpes zoster ophthalmicus.

Risk Period	Number of Ischemic Stroke Cases (*n* = 6,971)	Ischemic Stroke IR^a^ (95% CI)	Number of MI Cases (*n* = 3,946)	MI IR^a^ (95% CI)
**Baseline**	5,125	1	2,891	1
**Risk period after HZO**				
1 wk	93	2.73 (2.22–3.35)^b^	43	2.06 (1.52–2.79)^b^
2–4 wk	177	1.77 (1.52–2.05)^b^	85	1.38 (1.11–1.72)^b^
5–12 wk	326	1.29 (1.15–1.44)^b^	160	1.02 (0.87–1.20)
13–26 wk	428	1.06 (0.96–1.17)	282	1.15 (1.01–1.30)^c^
27–52 wk	651	1.02 (0.94–1.11)	421	1.07 (0.97–1.19)

^a^IRs age-adjusted in 2-y bands.

^b^ *p <* 0.005.

^c^ *p <* 0.05

Stratifying by zoster vaccination status revealed no evidence for a reduced IR for ischemic stroke during the first 4 wk after zoster diagnosis among individuals who received the zoster vaccine (*n* = 843) (IR 1.14, 95% CI 0.75–1.74) compared to unvaccinated individuals (*n* = 40,724) (IR 1.78, 95% CI 1.68–1.88) (*p*-value for interaction = 0.28). The overall IR combining vaccinated and unvaccinated individuals for the same 4-wk post-zoster period was 1.76 (95% CI 1.67–1.86). There was no evidence that the IR for MI after zoster diagnosis varied according to zoster vaccination status (*p* = 0.44): the IR in weeks 1–4 after zoster diagnosis was 1.36 (95% CI 0.78–2.39) in vaccinated individuals (*n* = 400) and 1.37 (95% CI 1.26–1.48) in unvaccinated individuals (*n* = 23,089), similar to the combined IR of 1.37 (95% CI 1.26–1.48) (Table 4).

**Table 4:** Age-adjusted incidence ratios for vascular events in risk periods after zoster diagnosis, stratified by vaccination status.

Outcome	Risk Period	Vaccinated^a^		Unvaccinated	
		Number of Cases	IR^b^ (95% CI)	Number of Cases	IR^b^ (95% CI)
**Ischemic stroke**		843		40,724	
	**Baseline**	602	1	30,412	1
	**Risk period after zoster**				
	1–4 wk	23	1.14 (0.75–1.74)	1,436	1.78 (1.68–1.88)
	5–12 wk	50	1.30 (0.97–1.74)	1,778	1.17 (1.11–1.23)
	13–26 wk	64	1.12 (0.86–1.46)	2,467	1.03 (0.99–1.07)
	27–52 wk	81	0.97 (0.76–1.24)	3,756	1.00 (0.97–1.04)
**MI**		400		23,089	
	**Baseline**	272	1	17,180	1
	**Risk period after zoster**				
	1–4 wk	13	1.36 (0.78–2.39)	668	1.37 (1.26–1.48)
	5–12 wk	18	1.04 (0.64–1.69)	992	1.07 (1.01–1.15)
	13–26 wk	37	1.49 (1.05–2.13)	1,466	1.01 (0.96–1.07)
	27–52 wk	46	1.21 (0.87–1.69)	2,329	1.02 (0.98–1.07)

Note that the analyses of MI excluded 167 vaccinated individuals whose MI or zoster occurred before the adjusted start date and 581 unvaccinated individuals whose MI occurred after the adjusted end date; the analyses of stroke excluded 370 vaccinated individuals whose stroke or zoster occurred before the adjusted start date and 1,017 unvaccinated individuals whose stroke occurred after the adjusted end date.

^a^ *p*-Values for interaction: stroke, *p* = 0.28; MI, *p* = 0.44.

^b^IRs age-adjusted in 2-y bands.

The secondary analysis of hemorrhagic stroke (*n* = 3,109 cases) indicated a similar pattern of increase and resolution of risk as for ischemic/nonspecific stroke, though less pronounced and with reduced precision because of the relatively few cases. The largest increase in hemorrhagic stroke rate, 1.6-fold, was observed in weeks 2–4 post-zoster (IR 1.61, 95% CI 1.29–2.02), reducing to 1.3-fold in weeks 5–12 (IR 1.30, 95% CI 1.10–1.53) and resolving thereafter.

Using the extension to the standard SCCS method to allow for nonrandom censoring of observation gave results virtually identical to those obtained in the primary analysis (S2 Table). Further sensitivity analyses excluding potentially fatal cases also gave similar findings (S3 Table).

## Discussion

This study demonstrates that zoster is associated with transiently increased rates of stroke and MI. The most marked increase was observed during the first week following zoster diagnosis, with a 2.4-fold increased rate of ischemic stroke (IR 2.37, 95% CI 2.17–2.59) and a 1.7-fold increased MI rate (IR 1.68, 95% CI 1.47–1.92), followed by a gradual reduction over 6 mo. The association we observed with MI is suggestive of a systemic association rather than one localized to the brain. A less marked association was observed for a secondary analysis of zoster and hemorrhagic stroke. Uptake of the zoster vaccine was low overall, with only 9% of participants receiving the vaccine during the study period. We found no evidence of a reduction in the IR for ischemic stroke or MI among vaccinees in the first 4 wk after zoster diagnosis, perhaps because of low patient numbers in the vaccinated groups, which limited the study’s power to assess this.

We have previously demonstrated, using UK general practice data and the SCCS method, that the rate of stroke is increased in the first 6 mo following zoster diagnosis, with the most marked increase being observed in the first 4 wk following zoster diagnosis and the rate gradually decreasing over time [8]. We did not assess the association between zoster and MI in our previous study, and the study period preceded the introduction of the zoster vaccine in the UK population. To our knowledge, all of the other studies that have assessed the risk of acute cardiovascular events following zoster have been limited by the potential for residual confounding due to the use of cohort designs and the inherent differences between individuals who develop zoster and those who do not develop zoster. Of these studies, two reported a 30% and a 4-fold increased stroke risk in the year following zoster and HZO, respectively, in a Taiwanese cohort in an administrative database [12,17]. Details about the timing of stroke after zoster were not available from these studies. A Danish registry cohort study identified an increased stroke risk during the first year following zoster, with the most pronounced increase being observed in the first 2 wk following zoster diagnosis, which is consistent with our observations in this current study [18]. A UK cohort study from general practice reported a long-term increased risk of transient ischemic attack and MI up to 24 y after acute zoster [9]. The possibility of residual confounding and the focus on long-term, rather than acute, cardiovascular events mean that the study provides limited information on the temporality of acute cardiovascular events following zoster.

Our study is a large, population-based cohort of older individuals in the US. Since 98% of the US population aged 65 y or greater are Medicare beneficiaries, the results of the study are reasonably generalizable to the older US population. Medicare data include high-quality information on demographics, clinical encounters, and prescription drugs. Recent studies have confirmed that stroke and MI diagnoses in Medicare have high positive predictive value and specificity [19,20]. Residual confounding is frequently an issue when using administrative data for research. However, as our analyses were within-person using the SCCS method, fixed confounders are inherently controlled for, and we adjusted finely for increasing age over the observation period. Residual confounding by unmeasured time-varying factors is unlikely to produce the effect estimates observed; such factors would need to be associated with both the timing of zoster and the vascular event and be present in a sufficiently large proportion of study participants to introduce material confounding. Such factors could plausibly include major life events or stress. Possible increased ascertainment of vascular events in periods of close monitoring after zoster could provide an alternative explanation for the associations seen. However, the scope for any such case-finding bias is limited given that ischemic stroke and acute MI are associated with significant morbidity, usually resulting in hospitalization, and hence are unlikely to go undetected. In addition to the standard SCCS analysis, we used an extended version that allowed for nonrandom censoring of the observation period. The results were unchanged using the extension, which suggests that any bias due to increased risk of death as a result of stroke or MI was avoided.

The observed increased risk of stroke and MI following zoster is likely to be explained by multiple biological mechanisms. Our primary hypothesis is that inflammation may lead to arterial thrombosis on a background of atherosclerosis. A hemodynamic mechanism is also a possible basis for the increased risk of stroke following zoster. Acute elevation of blood pressure [21] relating to zoster-associated pain or stress—or, alternatively, VZV-induced vasculopathy with arterial rupture or aneurysm formation [7]—could lead to an increased risk of hemorrhagic stroke in particular.

Medicare data are administrative data, so misclassification of exposures and outcomes is possible. However, provided the exposure and outcome are ascertained independently, any random misclassification in SCCS analyses would tend to bias findings towards the null [22]. We employed a strict definition of herpes zoster, requiring individuals to have received antiviral therapy to reduce misclassification from the possible use of “rule out” codes, whereby possible herpes zoster is coded as definite herpes zoster, as Medicare is an administrative data source [11,23]. Use of this definition limited our ability to study the role of antiviral therapy in stroke and MI rates after zoster. In addition, individuals who receive antiviral therapy are likely to have more severe zoster; hence, our study findings may be less applicable to individuals with mild zoster that would not warrant antiviral therapy. Uptake of zoster vaccine was low (9%) among study participants (all of whom had zoster), with only 3% receiving the vaccine prior to developing zoster (vaccine failures), and thus even with a very large data source, there was limited power to assess whether vaccination modifies the association between zoster and acute cardiovascular events.

In conclusion, herpes zoster was associated with increased rates of both stroke and MI, with a particularly marked increase in the first week following zoster diagnosis and tailing off over a period of 6 mo. The rapid increase in the rate of acute cardiovascular events after zoster diagnosis, followed by gradual resolution, is supportive of a causative association. Zoster vaccination did not appear to modify the association between zoster and ischemic stroke or MI; this finding requires further study due to low vaccination rates. Our findings provide useful information that may help to prevent acute cardiovascular events in older people.

## Supporting Information

S1 Data Form CMS data request form. (XLS)

S1 STROBE Checklist The study STROBE checklist. (PDF)

S1 Table Participant characteristics stratified by vaccination status. (DOCX)

S2 Table Allowing for nonrandom censoring of observation. (DOCX)

S3 Table Excluding potentially fatal cases. (DOCX)

## Abbreviations

CMS: Centers for Medicare & Medicaid Services
CPT: Current Procedural Terminology
CVD: cardiovascular disease
HMO: health maintenance organization
HZO: herpes zoster ophthalmicus
ICD-9-CM: International Classification of Diseases, Ninth Revision, Clinical Modification
IQR: interquartile range
IR: incidence ratio
MI: myocardial infarction
PHN: post-herpetic neuralgia
SCCS: self-controlled case series
VZV: varicella zoster virus
