# Supplementary material for: Acute Cardiovascular Events after Herpes Zoster: A Self-Controlled Case Series Analysis in Vaccinated and Unvaccinated Older Residents of the United States
Source: PLoS Med. 2015 Dec 15;12(12):e1001919. doi: 10.1371/journal.pmed.1001919 (PMC4682931; doi:10.1371/journal.pmed.1001919)
Supplement: S1 STROBE Checklist — (PDF) [file pmed.1001919.s002.pdf]

STROBE Statement—checklist of items that should be included in reports of observational studies

	Item No.	Recommendation	Section, Paragraph No.	Relevant text from manuscript
**Title and abstract**	1	(*a*) Indicate the study's design with a commonly used term in the title or the abstract	Title	Acute cardiovascular events after herpes zoster: a self-controlled case series analysis in vaccinated and unvaccinated older residents of the United States
		(*b*) Provide in the abstract an informative and balanced summary of what was done and what was found	Abstract	**Background** Herpes zoster is common and can have serious consequences. Additionally, emerging data suggest an increased risk of acute cardiovascular events following herpes zoster. However, to our knowledge, existing association studies compare outcomes between individuals, and are therefore vulnerable to between-person confounding. In this study, we used a within-person study design to quantify any short-term increased risk of acute cardiovascular events (stroke and myocardial infarction) after zoster and to assess whether zoster vaccination modifies this association. **Methods and Findings** The self-controlled case series method was used to estimate rates of stroke and acute myocardial infarction in defined periods after herpes zoster compared to other time periods, within individuals. Participants were fully eligible Medicare beneficiaries aged ≥65 years with a herpes zoster diagnosis and either an ischemic stroke (n=42,954) or myocardial infarction (n=24,237) between 1st January 2006 and 31st December 2011. Age-adjusted incidence ratios for stroke and myocardial infarction during pre-defined periods up to 12 months after zoster relative to unexposed time periods were calculated using conditional Poisson regression. We observed a marked increase in the rate of acute cardiovascular events in the first week after zoster: a 2.4-fold increased ischemic stroke rate (IR 2.37, 95% CI 2.17-2.59) and a 1.7-fold increased MI rate (IR 1.68, 95% CI 1.47-1.92), followed by a gradual

				resolution over six months. Zoster vaccination did not appear to modify the association with MI (interaction p value 0.44). We also found no evidence for a difference in the incidence ratios for ischemic stroke between vaccinated (IR 1.14, 95% CI 0.75-1.74) and unvaccinated (IR 1.78, 95% CI 1.68-1.88) individuals during the first four weeks after zoster (interaction p value 0.28). The relatively few vaccinated individuals limited the study's power to assess the role of vaccination. **Conclusions** Stroke and MI rates are transiently increased after exposure to herpes zoster. We found no evidence for a role for zoster vaccination in these associations. These findings enhance our understanding of the temporality and magnitude of the association between zoster and acute cardiovascular events.
**Introduction**				
Background/rationale	2	Explain the scientific background and rationale for the investigation being reported	Introduction, para 1-3	Strong and increasing evidence supports a period of increased cardiovascular risk shortly after exposure to specific infections, with rates of myocardial infarction (MI) and stroke increased five and three-fold respectively following acute respiratory infections.[1] The basis for increased cardiovascular events following acute infections is hypothesized to be due to endothelial dysfunction, characterized by atheromatous plaque rupture and the development of a prothrombotic environment.[2] As acute cardiovascular disease, specifically ischemic stroke and MI, are major causes of morbidity and mortality in the US and worldwide, understanding the basis for acute cardiovascular events and any potential for prevention becomes increasingly important.[3] Herpes zoster results from reactivation of dormant varicella zoster virus (VZV). Herpes zoster is an important disease as it affects one million Americans per year and is frequently complicated by prolonged, severe disabling pain, *post-herpetic neuralgia (PHN)*.[4,5] Zoster-associated morbidity has led to the introduction of a targeted vaccination program for individuals aged 60 years or greater in the US in 2006. The zoster

				vaccine has been shown to be effective in routine practice against incident zoster and PHN. Despite this, uptake of this vaccine has been disappointing (3.9%) following its introduction in the US, with important discrepancies in vaccine uptake by race and by income status.[6]<br>Recent studies have proposed that the risk of stroke may be increased in the year following an acute episode of herpes zoster, possibly also mediated by VZV replication in arterial walls resulting in cerebral vasculopathy.[7] Most studies assessing the association between herpes zoster and stroke have been limited by residual confounding as comparisons were made between individuals who developed herpes zoster and those who did not, and these individuals have important differences in underlying vascular risk that are difficult to measure and account for. Our group used the self-controlled case series (SCCS) method, which eliminates between-person confounding, to demonstrate an increased risk of stroke in the first six months following herpes zoster in the UK population and that antiviral therapy might lessen this association.[8] One UK cohort study, which may be limited by residual confounding, also suggested a longer-term increased risk of stroke and MI up to 24 years following acute herpes zoster.[9] To our knowledge, no previous study has determined the risk of MI in the period immediately following herpes zoster or assessed the role of zoster vaccination in the association between zoster and acute cardiovascular events.
Objectives	3	State specific objectives, including any prespecified hypotheses	Introduction, para 4	We used administrative claims data from older US Medicare beneficiaries to test the hypothesis of an increased risk of stroke and MI in periods shortly following acute episodes of herpes zoster and to assess whether zoster vaccination might modify this association.
**Methods**				
Study design	4	Present key elements of study design early in the paper	Methods: Study Design	The SCCS method was used to estimate the rate of acute cardiovascular events (stroke and MI) in defined periods after herpes zoster compared

				to other time periods, within individuals.[10] The SCCS approach overcomes the problem of between-person confounding inherent in other observational study designs by making within-person comparisons among individuals with zoster, whereby each case is his/her own control.
Setting	5	Describe the setting, locations, and relevant dates, including periods of recruitment, exposure, follow-up, and data collection	Methods: Data Source	This study used Medicare administrative claims data (Research Identifiable Files including Medicare Provider Analysis and Review (MEDPAR) (inpatient hospital and skilled nursing facilities), Outpatient, Carrier (physician/supplier) and Prescription Drug claims) for the period 1st January 2006 to 31st December 2011, obtained from the CMS.
Participants	6	(*a*) *Cohort study*—Give the eligibility criteria, and the sources and methods of selection of participants. Describe methods of follow-up<br>*Case-control study*—Give the eligibility criteria, and the sources and methods of case ascertainment and control selection. Give the rationale for the choice of cases and controls<br>*Cross-sectional study*—Give the eligibility criteria, and the sources and methods of selection of participants	Methods: Participants	The source population included all Medicare beneficiaries aged 65 years or older enrolled in Parts A (hospital insurance), B (supplemental medical insurance) and D (prescription drug coverage) who had both a zoster diagnosis and a Chronic Conditions Warehouse (CCW) flag for acute MI or stroke/transient ischemic attack (derived from the CMS's own algorithms) during the study period.<br>Participants were observed from the date they fulfilled the following criteria: i) 12 months continuous enrolment in Parts A and B, ii) eligible for Part D, and iii) not in a Health Maintenance Organization (HMO) (claims for beneficiaries enrolled in HMOs are not processed by CMS, hence their clinical data are incomplete). The 12 month minimum Part A/B enrolment criterion was applied to enable the study of incident rather than prevalent events. Observation was censored at the earliest of the date the participant died, joined a HMO, lost eligibility for part A, B or D, or the study period ended (31st December 2011).<br>We identified each participant's earliest recorded zoster episode and vascular event during the observation period and excluded individuals with evidence of vascular events or herpes zoster prior to the observation period. Diagnoses of incident events were extracted from Outpatient and Carrier (healthcare provider) files and from the primary diagnostic field in Inpatient files. Patients with only secondary inpatient diagnoses were excluded due to uncertainty of the timing of events that

				were not the primary reason for admission to hospital and hence may have occurred at any time during hospitalization. For the stroke analyses, we additionally excluded individuals with: i) subarachnoid hemorrhage (SAH) or established risk factors for SAH including cerebral aneurysms in the circle of Willis or arteriovenous malformations at any time during enrolment, and ii) encephalitis diagnoses recorded up to 12 months after the stroke which may represent encephalitis initially misclassified as stroke.
		(*b*) *Cohort study*—For matched studies, give matching criteria and number of exposed and unexposed<br>*Case-control study*—For matched studies, give matching criteria and the number of controls per case		N/A
Variables	7	Clearly define all outcomes, exposures, predictors, potential confounders, and effect modifiers. Give diagnostic criteria, if applicable	Methods: Exposure	**Exposure**<br>We defined incident herpes zoster as the presence of a specific International Classification of Diseases, Ninth Revision, Clinical Modification (ICD-9-CM) diagnostic code 053x (where x indicates the $4^{th}/5^{th}$ digits can take any value, excluding PHN codes 05312 and 05313) with an antiviral prescription in the seven days before or after diagnosis. The requirement for antiviral therapy has been shown to improve the positive predictive value of using zoster diagnosis codes to identify incident cases.[11] Herpes zoster ophthalmicus (HZO) was identified from ICD-9-CM codes 0532x recorded up to 12 months after the incident zoster code, or when zoster codes were nonspecific, either from acute eye infections or associated treatments within two weeks of the zoster diagnosis or from specific non-acute eye conditions associated with zoster, e.g. conjunctival scarring or episcleritis, recorded for the first time up to three months after zoster. HZO was analyzed separately as previous research suggested a markedly increased risk of stroke in this group.[12] In keeping with the primary zoster definition, an accompanying antiviral claim was also required for HZO.<br>Herpes zoster vaccination status was ascertained from records of Current Procedural Terminology (CPT) code 90736 and National Drug Codes

	for zoster vaccine purchase in the Part D drug files. Additionally, vaccine administration records up to seven days after purchase (CPT code 90471 or Healthcare Common Procedure Coding System (HCPCS) code G0377) were identified. We estimated the date of zoster vaccination as the earlier of the date of CPT code 90736 or date of administration. In the absence of a corresponding administration date and when no CPT code 90736 was recorded, the vaccine purchase date was used as a proxy (procedure/administration dates were considered better estimates of vaccination date than purchase dates).
Methods: Outcomes	**Outcomes**<br>We identified and classified acute cardiovascular events with specific ICD-9-CM codes for stroke (433x1, 434x1 (ischemic); 431, 4329 (hemorrhagic) and 436 (nonspecific)) and MI (410x, excluding codes with 5th digit 2 indicating a subsequent episode of care). As our primary interest was in acute thrombotic cardiovascular events (ischemic stroke and MI), we excluded hemorrhagic strokes from the primary analysis. To do this, we created stroke episodes whereby successive stroke codes within 28 days of each other were assumed to correspond to the same episode to differentiate stroke subtypes (ischemic, hemorrhagic or unspecified). For episodes containing stroke codes of different types, we used the following hierarchy for classification: hemorrhagic codes took precedence (based on the assumption that hemorrhagic strokes are coded more accurately than ischemic strokes) and ischemic codes trumped nonspecific codes. Because hemorrhagic strokes could potentially result from arterial rupture or aneurysm following VZV vasculopathy, or from a transient spike in blood pressure (for example, in response to zoster-associated pain), we undertook (in response to peer review) a secondary analysis of hemorrhagic strokes. To maintain an important assumption of the case series method – that recurrent outcome events within individuals are independent – we restricted all analyses to participants' first stroke or MI in the observation period.

			Methods: Comorbidities and demographics	**Comorbidities and demographics**<br>For descriptive purposes, pre-existing cardiovascular disease (CVD) (MI, stroke, transient ischemic attack, ischemic heart disease, heart failure or atrial fibrillation) and CVD risk factors (hypertension, hyperlipidemia, diabetes, chronic kidney disease or chronic obstructive pulmonary disease) were identified using CCW flags indicating the earliest occurrence of each condition. The number of unique prescriptions an individual received in the 12 months before their vascular event (as an indicator of overall poorer health) was determined by identifying all unique prescriptions and excluding duplicate records with the same product code or the same generic and brand names on the same date. Ethnicity was defined according to the Social Security Administration's master beneficiary record (based on self-report) and categorized into white, black, Asian, Hispanic and other/unknown. "State buy-in", whereby the state pays Medicare premiums for low-income individuals, was used as a proxy for low income. Current age was derived from date of birth and included in all analyses as a time-varying covariate.
Data sources/ measurement	8*	For each variable of interest, give sources of data and details of methods of assessment (measurement). Describe comparability of assessment methods if there is more than one group	Methods: Participants, para 2;	Diagnoses of incident events were extracted from Outpatient and Carrier (healthcare provider) files and from the primary diagnostic field in Inpatient files. Patients with only secondary inpatient diagnoses were excluded due to uncertainty of the timing of events that were not the primary reason for admission to hospital and hence may have occurred at any time during hospitalization.
			Exposure, para 2	Herpes zoster vaccination status was ascertained from records of Current Procedural Terminology (CPT) code 90736 and National Drug Codes for zoster vaccine purchase in the Part D drug files. Additionally, vaccine administration records up to seven days after purchase (CPT code 90471 or Healthcare Common Procedure Coding System (HCPCS) code G0377) were identified.

Bias	9	Describe any efforts to address potential sources of bias	Methods: Study Design, para 1;	The SCCS approach overcomes the problem of between-person confounding inherent in other observational study designs by making within-person comparisons among individuals with zoster, whereby each case is his/her own control.
			Participants, para 2;	Patients with only secondary inpatient diagnoses were excluded due to uncertainty of the timing of events that were not the primary reason for admission to hospital and hence may have occurred at any time during hospitalization.
			Outcomes, para 1;	To maintain an important assumption of the case series method – that recurrent outcome events within individuals are independent – we restricted all analyses to participants' first stroke or MI in the observation period.
			Analysis, para 1	Incidence ratios (IRs) for stroke and MI during pre-defined periods after exposure to zoster relative to unexposed time periods were calculated using conditional Poisson regression, adjusting for age in two-year bands. We defined a 12-month exposed period starting the day after zoster, subdivided into five risk windows: week 1, weeks 2-4, 5-12, 13-26 and 27-52 post-zoster. When calculating the IR for any given risk window, the four remaining risk windows were still considered "exposed". All other observation time made up the baseline (unexposed) period, with the exception of the day of zoster and the four weeks before zoster. The day of zoster was excluded as MI or stroke records on the same day as incident zoster may represent retrospective diagnoses. We excluded four weeks prior to zoster as vascular risk during this pre-zoster period may be higher or lower than in other unexposed periods because the chance of developing and/or presenting with zoster shortly after a vascular event may differ from other time periods. Removing these periods from baseline was an attempt to avoid bias in the effect estimates that could have operated in either direction depending on the variation in vascular risk.

			Analysis, para 3	To allow for non-random censoring of observation due to death as a result of a vascular event (which may lead to bias if uncorrected), an extension to the standard SCCS method was used.[15]
Study size	10	Explain how the study size was arrived at	Methods: Participants, para 1	The source population included all Medicare beneficiaries aged 65 years or older enrolled in Parts A (hospital insurance), B (supplemental medical insurance) and D (prescription drug coverage) who had both a zoster diagnosis and a Chronic Conditions Warehouse (CCW) flag for acute MI or stroke/transient ischemic attack (derived from the CMS's own algorithms) during the study period.
Quantitative variables	11	Explain how quantitative variables were handled in the analyses. If applicable, describe which groupings were chosen and why	Methods: Comorbidities and demographics;	Current age was derived from date of birth and included in all analyses as a time-varying covariate.
			Analysis, para 1	…adjusting for age in two-year bands.
Statistical methods	12	(*a*) Describe all statistical methods, including those used to control for confounding	Methods: Analysis, para 1	Incidence ratios (IRs) for stroke and MI during pre-defined periods after exposure to zoster relative to unexposed time periods were calculated using conditional Poisson regression, adjusting for age in two-year bands. We defined a 12-month exposed period starting the day after zoster, subdivided into five risk windows: week 1, weeks 2-4, 5-12, 13-26 and 27-52 post-zoster. When calculating the IR for any given risk window, the four remaining risk windows were still considered "exposed". All other observation time made up the baseline (unexposed) period, with the exception of the day of zoster and the four weeks before zoster.
			Analysis, para 2	The primary analysis assessed the association between zoster (all cases, irrespective of site) and ischemic stroke and MI separately. Additionally, the association between HZO and each outcome was assessed. A secondary analysis of hemorrhagic strokes (excluded from the primary analysis) was conducted to examine the association between zoster and this stroke sub-type.

		Analysis, para 4	Standard SCCS analyses were undertaken using STATA, version 13 (StataCorp, College Station, Texas); modified analyses accounting for event-dependent censoring were performed in R, version 3.0.2.
	(*b*) Describe any methods used to examine subgroups and interactions	Methods: Analysis, para 2	We undertook further analyses to explore whether vascular risk after zoster was different in those who received the zoster vaccine and those who did not. We defined vaccinated individuals as those who received the zoster vaccine before they developed zoster. To allow for effective immunization, these individuals were classified as vaccinated from 30 days after receipt of the vaccine.[13] Hence for these stratified analyses, the adjusted start date for vaccinated individuals was from the later of this date and their original start date. "Unvaccinated" individuals comprised those who did not receive the zoster vaccine and additionally, those who were vaccinated after their incident zoster episode and whose observation was thus curtailed at vaccination. To allow for small numbers in the vaccinated strata, the first two risk windows post-zoster (week 1, weeks 2-4) were combined.
	(*c*) Explain how missing data were addressed		N/A
	(*d*) *Cohort study*—If applicable, explain how loss to follow-up was addressed<br>*Case-control study*—If applicable, explain how matching of cases and controls was addressed<br>*Cross-sectional study*—If applicable, describe analytical methods taking account of sampling strategy	Methods: Participants, para 1	Observation was censored at the earliest of the date the participant died, joined a HMO, lost eligibility for part A, B or D, or the study period ended (31st December 2011).
	(*e*) Describe any sensitivity analyses	Methods: Analysis, para 3	To allow for non-random censoring of observation due to death as a result of a vascular event (which may lead to bias if uncorrected), an extension to the standard SCCS method was used.[15] In addition, the primary analysis was repeated excluding individuals who died or whose observation ended within 90 days after their vascular event (possibly indicating death).
**Results**			

Participants	13*	(a) Report numbers of individuals at each stage of study—eg numbers potentially eligible, examined for eligibility, confirmed eligible, included in the study, completing follow-up, and analysed	Results, para 1	The initial study population comprised 351,865 individuals, of whom 42,954 zoster cases with incident ischemic stroke and 24,237 with acute MI fulfilled the eligibility criteria and were included in the primary analysis (**Fig 2**).
		(b) Give reasons for non-participation at each stage		See **Fig 2**. Identification of study participants
		(c) Consider use of a flow diagram		See **Fig 2**.
Descriptive data	14*	(a) Give characteristics of study participants (eg demographic, clinical, social) and information on exposures and potential confounders	Results, para 1-2	Characteristics of these individuals are presented in **Table 1**. The median age at zoster was 80 years (interquartile range (IQR) 74-86 years) and median observation period was 5 years (IQR 4-5 years). The majority of participants were female (71% of zoster cases with stroke, 64% of MIs) and white (88% of strokes, 90% of MIs); 5% were black, the remaining 6% were Asian (2%), Hispanic (2%) or of other/unknown ethnicity (2%). 16% of zoster cases had HZO, the remaining 84% had zoster of an unspecified site. 34% of cases were of low income and 90% had evidence of pre-existing cardiovascular disease (CVD) before zoster.<br>A small minority of cases received the zoster vaccine before developing zoster (3% of cases with stroke, 2% of MIs), 6% received the vaccine after zoster and 91% were unvaccinated throughout the observation period. Characteristics of individuals included in the analyses stratified by zoster vaccination status are given in **S1 Table** in the Supplement.
		(b) Indicate number of participants with missing data for each variable of interest		See **Table 1**. Participant characteristics
		(c) *Cohort study*—Summarise follow-up time (eg, average and total amount)	Results, para 1	…median observation period was 5 years (IQR 4-5 years).
Outcome data	15*	*Cohort study*—Report numbers of outcome events or summary measures over time	Results, para 1	42,954 zoster cases with incident ischemic stroke and 24,237 with acute MI fulfilled the eligibility criteria and were included in the primary analysis.<br>*Note that this study is a self-controlled case series study using data from exposed cases only (individuals with both the exposure (herpes zoster) and the outcome (an acute vascular event). Hence all participants*

				experienced an outcome event and were exposed during the study period.
		Case-control study—Report numbers in each exposure category, or summary measures of exposure		N/A
		Cross-sectional study—Report numbers of outcome events or summary measures		N/A
Main results	16	(*a*) Give unadjusted estimates and, if applicable, confounder-adjusted estimates and their precision (eg, 95% confidence interval). Make clear which confounders were adjusted for and why they were included	Results, para 3 & Table 2	The rate of ischemic stroke was significantly increased up to three months after zoster (any site) compared to the baseline rate: the most marked increase, by 2.4-fold, was observed within the first week (IR 2.37, 95% CI 2.17-2.59), reducing to 1.6-fold in weeks 2-4 (IR 1.55, 95% CI 1.46-1.66), 1.2-fold in weeks 5-12 (IR 1.17, 95% CI 1.11-1.22), and resolving over the subsequent three months (weeks 13-26: IR 1.03, 95% CI 0.99-1.07; weeks 27-52: IR 1.00, 95% CI 0.96-1.03). A similar though less marked association was observed for MI in the three months post-zoster, with a 68% increased MI rate in the first week (IR 1.68, 95% CI 1.47-1.92) compared to baseline, and a similar pattern of resolution as for stroke (weeks 2-4: IR 1.25, 95% CI 1.14-1.37; weeks 5-12: IR 1.07, 95% CI 1.00-1.14; weeks 13-26: IR 1.02, 95% CI 0.96-1.07; weeks 27-52: IR 1.02, 95% CI 0.98-1.07)<br>See **Table 2.** Primary analysis: Age-adjusted incidence ratios for stroke and myocardial infarction (MI) in risk periods after zoster.
		(*b*) Report category boundaries when continuous variables were categorized		See **Table 2**.
		(*c*) If relevant, consider translating estimates of relative risk into absolute risk for a meaningful time period		N/A
Other analyses	17	Report other analyses done—eg analyses of subgroups and interactions, and sensitivity analyses	Results, para 4;<br>Tables 3 & 4	Analyses restricted to cases with HZO (n=6,971 with ischemic stroke, 3,946 with MI) yielded comparable associations as the primary analysis (week 1 post-HZO: stroke IR 2.73, 95% CI 2.22-3.35; MI IR 2.06, 95% CI 1.52-2.79) that resolved over the same time period.<br>See **Table 3.** Age-adjusted incidence ratios for stroke and myocardial infarction (MI) in risk periods after Herpes zoster ophthalmicus.

			Results, para 5	Stratifying by zoster vaccination status revealed no evidence for a reduced IR for ischemic stroke during the first four weeks after zoster among individuals who received the zoster vaccine (n=843) (IR 1.14, 95% CI 0.75-1.74) compared to unvaccinated individuals (n=40,724) (IR 1.78, 95% CI 1.68-1.88), (p value for interaction=0.28). The overall IR combining vaccinated and unvaccinated individuals for the same four-week post-zoster period was 1.76, 95% CI 1.67-1.86. There was no evidence that the IRs for MI after zoster varied according to zoster vaccination status (p=0.44): the IR in weeks 1-4 post-zoster was 1.36, 95% CI 0.78-2.39 in vaccinated individuals (n=400) and 1.37, 95% CI 1.26–1.48 in unvaccinated individuals (n=23,089), similar to the combined IR of 1.37, 95% CI 1.26-1.48<br>See **Table 4.** Age-adjusted incidence ratios for vascular events in risk periods after zoster, stratified by vaccination status.
			Results, para 6-7	The secondary analysis of hemorrhagic stroke (n=3109 cases) indicated a similar pattern of increase and resolution of risk as for ischemic/nonspecific stroke, though less pronounced and with reduced precision due to the relatively few cases. The largest increase in hemorrhagic stroke rate, by 1.6-fold, was observed in weeks 2-4 post-zoster (IR 1.61, 95% CI 1.29-2.02), reducing to 1.3-fold in weeks 5-12 (IR 1.30, 95% CI 1.10-1.53) and resolving thereafter.<br>Using the extension to the standard SCCS method to allow for non-random censoring of observation gave virtually identical results to those obtained in the primary analysis (**S2 Table**). Further sensitivity analyses excluding potentially fatal cases also gave similar findings (**S3 Table**).
**Discussion**				
Key results	18	Summarise key results with reference to study objectives	Discussion, para 1	This study demonstrates that acute zoster is associated with transiently increased rates of stroke and MI. The most marked increase was observed during the first week following zoster with a 2.4-fold increased rate of ischemic stroke (IR 2.37, 95% CI 2.17-2.59) and a 1.7-fold increased MI rate (IR 1.68, 95% CI 1.47-1.92) followed by a gradual reduction over six months.

				Uptake of the zoster vaccine was low overall with only 9% of participants receiving the vaccine during the study period. We found no evidence for a reduction in the IRs for ischemic stroke or MI among vaccinees in the first four weeks after zoster..
Limitations	19	Discuss limitations of the study, taking into account sources of potential bias or imprecision. Discuss both direction and magnitude of any potential bias	Discussion, para 3-4	Residual confounding is frequently an issue when using administrative data for research. However, as our analyses were within-person using the SCCS method, fixed confounders are inherently controlled for and we adjusted finely for increasing age over the observation period. Residual confounding by unmeasured time-varying factors is unlikely to produce the effect estimates observed; such factors would need to be associated with both the timing of zoster and the vascular event and be present in a sufficiently large proportion of study participants to introduce any material confounding. Such a factor could plausibly include major life events or stress.<br>Medicare data are administrative data so misclassification of exposures and outcomes is possible. However, provided the exposure and outcome are ascertained independently, any such random misclassification in SCCS analyses would tend to bias findings towards the null.[22] We employed a strict definition of herpes zoster, requiring individuals to have received antiviral therapy to reduce misclassification from the possible use of "rule out" codes, whereby possible herpes zoster is coded as definite herpes zoster, as Medicare is an administrative data source.[11,23] Use of this definition limited our ability to study the role of antiviral therapy in stroke and MI rates after zoster.<br>Uptake of zoster vaccine was low (9%) among study participants (all of whom had zoster), with only 3% receiving the vaccine prior to developing zoster (the vaccine failures), and thus even with a very large data source there was limited power to assess whether vaccination modifies the association between zoster and acute cardiovascular events.
Interpretation	20	Give a cautious overall interpretation of results considering objectives, limitations, multiplicity of analyses, results from similar studies, and other relevant evidence	Discussion, para 1<br>Discussion, para 2	The association we observed with MI is suggestive of a systemic association rather than one localized to the brain.<br>We have previously demonstrated, using UK general practice data and the SCCS method, that the rate of stroke is increased in the first six months

		following zoster, with the most marked increase being observed in the first four weeks following zoster and gradually reducing over time.[8] We did not assess the association between zoster and MI in our previous study and the study period preceded the introduction of the zoster vaccine in the UK population.<br>To our knowledge, all of the other studies that have assessed the risk of acute cardiovascular events following zoster have been limited by the potential for residual confounding due to the use of cohort designs and the inherent differences between individuals who develop zoster and those who do not develop zoster. Of these studies, two reported a 30% and a fourfold increased stroke risk in the year following zoster and HZO respectively in a Taiwanese cohort in an administrative database.[12,17] Details about the timing of stroke following zoster were not available from these studies. A Danish registry cohort study identified an increased stroke risk during the first year following zoster with the most pronounced increase being observed in the first two weeks following zoster, which is consistent with our observations in this current study.[18]
	Discussion, para 4	The observed increased risk of stroke and MI following zoster is likely to be explained by multiple biological mechanisms. Our primary hypothesis was that inflammation may lead to arterial thrombosis on a background of atherosclerosis. A hemodynamic mechanism is also a possible basis for the increased risk of stroke following zoster. Acute elevation of blood pressure[21] relating to zoster-associated pain or stress, or alternatively VZV-induced vasculopathy with arterial rupture or aneurysm formation[7], could lead to increased risk of hemorrhagic strokes in particular.
	Discussion, para 6	In conclusion, herpes zoster was associated with increased rates of both stroke and MI with a particularly marked increase in the first week following zoster, tailing off over a period of six months. The rapid increase in the rate of acute cardiovascular events followed by gradual resolution is supportive of a causative association. Zoster vaccination did not appear to modify the associations between zoster and ischemic stroke or MI; this finding requires further study due to low vaccination rates.

Generalisability	21	Discuss the generalisability (external validity) of the study results	Discussion, para 3	Our study is a large, population-based cohort of older individuals in the US. 98% of the US population aged 65 years or greater are Medicare beneficiaries; hence the results of the study are reasonably generalizable to the older US population.
			Discussion, para 5	… individuals who received antiviral therapy are likely to have had more severe zoster, hence study findings may be less applicable to individuals with mild zoster that would not warrant antiviral therapy.
**Other information**				
Funding	22	Give the source of funding and the role of the funders for the present study and, if applicable, for the original study on which the present article is based	Funding	This work was supported by a Wellcome Trust Senior Fellowship in Clinical Science (to LS, grant number: 098504/Z/12/Z), an NIHR Clinician Scientist Fellowship (to SML, grant number: NIHR/CS/010/014), an NIHR Career Development Fellowship (to SLT, grant number: NIHR/CDF/2010-03-32) and a grant from the Stroke Association (grant number: TSA 2011/05). The funders had no role in study design, data collection and analysis, decision to publish, or preparation of the manuscript.

*Give information separately for cases and controls in case-control studies and, if applicable, for exposed and unexposed groups in cohort and cross-sectional studies.

Note: An Explanation and Elaboration article discusses each checklist item and gives methodological background and published examples of transparent reporting. The STROBE checklist is best used in conjunction with this article (freely available on the Web sites of PLoS Medicine at http://www.plosmedicine.org/, Annals of Internal Medicine at http://www.annals.org/, and Epidemiology at http://www.epidem.com/). Information on the STROBE Initiative is available at www.strobe-statement.org.
