# Supplementary material for: Acute Cardiovascular Events after Herpes Zoster: A Self-Controlled Case Series Analysis in Vaccinated and Unvaccinated Older Residents of the United States
Source: PLoS Med. 2015 Dec 15;12(12):e1001919. doi: 10.1371/journal.pmed.1001919 (PMC4682931; doi:10.1371/journal.pmed.1001919)
Supplement: S1 Table — (DOCX) [file pmed.1001919.s003.docx]

**S1 Table.** Participant characteristics stratified by vaccination status.

| **Characteristic** | **Zoster Cases with Ischemic Stroke** | | **Zoster Cases with MI** | |
| --- | --- | --- | --- | --- |
|  | Vaccinated  n=843 | Unvaccinated n=40724 | Vaccinated  n=400 | Unvaccinated n=23089 |
| **HZO** | 121 (14.4) | 6624 (16.3) | 70 (17.5) | 3773 (16.3) |
| **Age at zoster diagnosis (years)** | 80.4 (75.0-85.2) | 80.5 (74.4-86.0) | 79.4 (73.0-84.3) | 79.7 (73.6-85.6) |
| 65-69 | 67 (7.9) | 4094 (10.0) | 40 (10.0) | 2720 (11.8) |
| 70-79 | 338 (40.1) | 15435 (37.9) | 168 (42.0) | 9087 (39.4) |
| 80-89 | 385 (45.7) | 16651 (40.9) | 164 (41.0) | 8849 (38.3) |
| ≥90 | 53 (6.3) | 4544 (11.2) | 28 (7.0) | 2433 (10.5) |
| **Age at HZO diagnosis^a^ (years)** | 80.3 (74.7-84.6) | 81.1 (75.0-86.4) | 77.4 (72.7-84.1) | 80.3 (74.2-86.2) |
| **Gender** |  |  |  |  |
| Male | 268 (31.8) | 12027 (29.5) | 132 (33.0) | 8233 (35.7) |
| Female | 575 (68.2) | 28697 (70.5) | 268 (67.0) | 14856 (64.3) |
| **Ethnicity** |  |  |  |  |
| White | 766 (90.9) | 35891 (88.1) | 360 (90.0) | 20625 (89.3) |
| Black | ^b^ | 2293 (5.6) | ^b^ | 1136 (4.9) |
| Asian | 53 (6.3) | 865 (2.1) | 19 (4.7) | 428 (1.9) |
| Hispanic | ^b^ | 1021 (2.5) | ^b^ | 546 (2.4) |
| Other/Unknown | ^b^ | 654 (1.6) | ^b^ | 354 (1.5) |
| **Low income^c^** | 134 (15.9) | 14318 (35.2) | 62 (15.5) | 8047 (34.9) |
| **Number of prescriptions in 12 mo before vascular event** | 43 (25-67) | 48 (28-77) | 46 (26-72) | 51 (29-81) |
| **Quintiles of the distribution of number of prescriptions** |  |  |  |  |
| **1** | 181 (21.5) | 7972 (19.6) | 93 (23.3) | 4506 (19.5) |
| **2** | 198 (23.5) | 8179 (20.1) | 80 (20.0) | 4668 (20.2) |
| **3** | 187 (22.2) | 8047 (19.8) | 94 (23.5) | 4589 (19.9) |
| **4** | 163 (19.3) | 8244 (20.2) | 80 (20.0) | 4651 (20.1) |
| **5** | 114 (13.5) | 8282 (20.3) | 53 13.2) | 4675 (20.3) |
| **CVD before zoster^d^** | 745 (88.4) | 36627 (89.9) | 362 (90.5) | 20989 (90.9) |
| **Risk factor for CVD before zoster^e^** | 832 (98.7) | 40038 (98.3) | 398 (99.5) | 22779 (98.7) |
| **Total observation (years)** | 5.0 (4.6-5.0) | 5.0 (4.0-5.0) | 5.0 (4.1-5.0) | 5.0 (3.7-5.0) |
| **Died or follow-up ended ≤90 days after vascular event** | 71 (8.4) | 4039 (9.9) | 57 (14.3) | 3762 (16.3) |

Data are given as n (percent) or median (IQR)

^a^121 vaccinated and 6624 unvaccinated stroke cases; 70 vaccinated and 3773 unvaccinated MI cases

^b^Data suppressed to remain compliant with CMS’s small-sized-cell privacy policy

^c^State buy-in at any time during enrolment

^d^MI, stroke, transient ischemic attack, ischemic heart disease, heart failure, or atrial fibrillation

^e^Hypertension, hyperlipidemia, diabetes, chronic kidney disease, or chronic obstructive pulmonary disease
