# Supplementary material for: Acute Cardiovascular Events after Herpes Zoster: A Self-Controlled Case Series Analysis in Vaccinated and Unvaccinated Older Residents of the United States
Source: PLoS Med. 2015 Dec 15;12(12):e1001919. doi: 10.1371/journal.pmed.1001919 (PMC4682931; doi:10.1371/journal.pmed.1001919)
Supplement: S2 Table — (DOCX) [file pmed.1001919.s004.docx]

**S2 Table.** Allowing for nonrandom censoring of observation: Age-adjusted incidence ratios for ischemic stroke and myocardial infarction in risk periods after zoster diagnosis.

| Risk period | Number of Ischemic Stroke Cases (n=42954) | Ischemic stroke IR^a^ (95% CI) | Number of MI Cases (n=24237) | MI IR^a^ (95% CI) |
| --- | --- | --- | --- | --- |
| Baseline | 32179 | 1 | 18071 | 1 |
| Risk period after zoster: |  |  |  |  |
| 1 wk | 499 | 2.37 (2.17-2.59) | 213 | 1.68 (1.47-1.93) |
| 2-4 wk | 967 | 1.55 (1.46-1.66) | 470 | 1.25 (1.14-1.37) |
| 5-12 wk | 1841 | 1.16 (1.11-1.22) | 1019 | 1.06 (1.00-1.13) |
| 13-26 wk | 2588 | 1.03 (0.98-1.07) | 1537 | 1.01 (0.96-1.06) |
| 27-52 wk | 3981 | 0.99 (0.96-1.02) | 2459 | 1.01 (0.97-1.05) |

^a^IRs age-adjusted in 2-year bands
