# Supplementary material for: Acute Cardiovascular Events after Herpes Zoster: A Self-Controlled Case Series Analysis in Vaccinated and Unvaccinated Older Residents of the United States
Source: PLoS Med. 2015 Dec 15;12(12):e1001919. doi: 10.1371/journal.pmed.1001919 (PMC4682931; doi:10.1371/journal.pmed.1001919)
Supplement: S3 Table — (DOCX) [file pmed.1001919.s005.docx]

**S3 Table.** Excluding potentially fatal cases: Age-adjusted incidence ratios for ischemic stroke and myocardial infarction in risk periods after zoster.

| Risk period | Number of Ischemic Stroke Cases (n=38650) | Ischemic stroke IR^a^ (95% CI) | Number of MI Cases (n=20251) | MI IR^a^ (95% CI) |
| --- | --- | --- | --- | --- |
| Baseline | 29538 | 1 | 15630 | 1 |
| Risk period after zoster: |  |  |  |  |
| 1 wk | 423 | 2.36 (2.14-2.60) | 149 | 1.54 (1.31-1.80) |
| 2-4 wk | 823 | 1.55 (1.44-1.66) | 341 | 1.19 (1.07-1.32) |
| 5-12 wk | 1528 | 1.12 (1.07-1.18) | 757 | 1.02 (0.95-1.10) |
| 13-26 wk | 2170 | 0.99 (0.95-1.04) | 1121 | 0.94 (0.88-1.00) |
| 27-52 wk | 3336 | 0.96 (0.93-1.00) | 1816 | 0.95 (0.91-1.00) |

^a^IRs age-adjusted in 2-year bands
